# Efficient spiking convolutional neural networks accelerator with multi-structure compatibility

**DOI:** 10.3389/fnins.2025.1662886

**Published:** 2025-09-26

**Authors:** Jiadong Wu, Lun Lu, Yinan Wang, Zhiwei Li, Changlin Chen, Qingjiang Li, Kairang Chen

**Affiliations:** ^1^College of Electronic Science and Technology, National University of Defense Technology, Changsha, China; ^2^College of Electronics and Internet of Things, Chongqing Polytechnic University of Electronic Technology, Chongqing, China

**Keywords:** spiking neural networks, spiking convolutional neural networks, artificial neural networks, brain-like computing, hardware accelerator, FPGA

## Abstract

Spiking Neural Networks (SNNs) possess excellent computational energy efficiency and biological credibility. Among them, Spiking Convolutional Neural Networks (SCNNs) have significantly improved performance, demonstrating promising applications in low-power and brain-like computing. To achieve hardware acceleration for SCNNs, we propose an efficient FPGA accelerator architecture with multi-structure compatibility. This architecture supports both traditional convolutional and residual topologies, and can be adapted to diverse requirements from small networks to complex networks. This architecture uses a clock-driven scheme to perform convolution and neuron updates based on the spike-encoded image at each timestep. Through hierarchical pipelining and channel parallelization strategies, the computation speed of SCNNs is increased. To address the issue of current accelerators only supporting simple network, this architecture combines configuration and scheduling methods, including grouped reuse computation and line-by-line multi-timestep computation to accelerate deep networks with lots of channels and large feature map sizes. Based on the proposed accelerator architecture, we evaluated two scales of networks, named small-scale LeNet and deep residual SCNN, for object detection. Experiments show that the proposed accelerator achieves a maximum recognition speed of 1, 605 frames/s at a 100 MHz clock for the LeNet network, consuming only 0.65 mJ per image. Furthermore, the accelerator, combined with the proposed configuration and scheduling methods, achieves acceleration for each residual module in the deep residual SCNN, reaching a processing speed of 2.59 times that of the CPU with a power consumption of only 16.77% of the CPU. This demonstrates that the proposed accelerator architecture can achieve higher energy efficiency, compatibility, and wider applicability.

## 1 Introduction

Artificial neural networks (ANNs) have achieved revolutionary applications in numerous fields, but their structure and capabilities still have a significant gap compared to real biological neural networks, especially in terms of the high energy consumption required during their computational processes (Javanshir et al., 2022). Compared to traditional ANNs, spiking neural networks (SNNs) have a higher degree of biomimicry to the human brain. SNNs express information through spike sequences. This enables SNNs to process information in both temporal and spatial domains, and to achieve a closer fit to neural dynamics models (Deng et al., 2020; Taherkhani et al., 2020). Due to the lack of effective learning algorithms, in its early development, SNNs had poor performance in multi-layer network structures, and related researches were also limited to shallow networks with multilayer perceptron (MLP) topological structures. However, the development and prosperity of deep neural networks in the past decade have had a significant impact on SNN research. Many structures, learning algorithms and ideas of ANNs have been successively introduced into SNNs. Among them, SNNs including convolution structure, namely, spiking convolutional neural networks (SCNNs), as well as learning algorithms such as ANN conversion method and backpropagation method, have gradually become the hotspots in the current research of SNNs. SCNNs are widely recognized to be able to achieve deeper network layers with higher performance like convolutional neural networks (CNNs). Sengupta et al. (2019) successfully trained the SCNNs of VGG architecture and even ResNet architecture through the method of ANN conversion to SNN. Spatio-temporal backpropagation (STBP) is a back-propagation algorithm effectively applied to multi-layer SCNN direct training (Wu et al., 2018, 2019); based on the STBP method (Zheng et al., 2021), threshold-dependent batch normalization (tdBN) is proposed to implement direct training of ResNet-scale SCNNs. In general, the current SCNNs are gradually approaching the existing deep ANNs in terms of network layers and scale. And the network performance and application potential of SCNNs in real-world scenarios have been significantly improved. However, this also means that more and more computing resources are needed for SCNNs, and the demand for efficiently running SCNNs is also increasing.

To improve the arithmetic speed of SNNs, various neuromorphic hardware accelerators have been proposed according to the characteristics of SNNs (Basu et al., 2022; Qu et al., 2022), such as BrainScaleS (Pehle et al., 2022) from Heidelberg University, TrueNorth (Merolla et al., 2014) from IBM, Loihi (Davies et al., 2018) from Intel and so on. At present, the network on chip (NoC) based multicore architecture is widely used in this kind of neuromorphic hardware accelerators, which is adopted to configure and simulate a large number of neurons and synapses (Nguyen et al., 2021). The information is passed inside the architecture through asynchronous event flows. Such architectures are more often used for deploying SNNs based on MLP structures, and are mainly used in the research of neuroscience and brain science. From a practical application perspective, even if some of these chips support running convolutional operations, the architecture of these neuromorphic chips can't make full use of hardware resources to speed up SCNNs with convolution operations due to the incomplete match in terms of computer mechanisms and network architectures (Zhang et al., 2021). In addition, with the increase of SCNN layers, ASICs with this kind of architecture require more complex neuron interconnection and asynchronous event handling mechanisms, which will constrain the hardware development cycle. Moreover, ASICs have long development cycles and low flexibility, making it difficult for ASIC-based accelerators to adapt to SCNNs, which is rapidly developing and iterating.

To address the above issues, some studies have designed corresponding hardware accelerators for SCNNs. Zhang et al. (2021) proposed a scalable, cost-efficient, and high-speed very large-scale integration (VLSI) architecture by performing pipelined convolutional operations on the snapshot of binary spike maps at each time-step. Kang et al. (2019) used the events stream with address event representation (AER) as the input for convolutional operations and designed an SCNN inference accelerator based on this. Ye et al. (2023) used an extended predictive correction (EPC) optimization method to design the circuit of LIF neurons to reduce computational complexity and hardware resources, and based on this neuron circuit, they designed an SNN accelerator supporting both MLP and CNN topologies. These accelerators are implemented based on FPGAs. The flexible and reconfigurable characteristics of FPGAs are more suitable for rapidly iterating SNNs. However, the above accelerators still have many shortcomings: existing SCNN FPGA accelerators are mainly designed for running SNNs with MLP or traditional convolutional topologies, but they lack effective support for different convolutional types such as residual convolutional networks; moreover, most existing accelerators can only run simple, fixed network structures and lack efficient support solutions for deep networks with large numbers of channels and feature map sizes (e.g., networks for object detection), severely limiting the application of accelerators in practical tasks.

To solve the above problems, a multi-structure compatible SCNN hardware architecture is proposed in this paper for more efficient acceleration of SCNNs. In response to the problem that existing neuromorphic chips cannot well match the convolutional topology of SCNNs, the architecture is based on a clock-driven design, which through the current spike image at each timestep calculates the convolutional results and updates the neuron state. This combines the spiking information format of SNNs with convolutional operations, simplifying the control logic of convolution. The accelerator is implemented on an FPGA and combines channel-wise parallel and pipelined structures to achieve efficient acceleration. Addressing the limited support for convolutional topologies in existing SCNN accelerators, the proposed accelerator architecture supports both traditional convolutional topologies and residual convolutional topologies. Furthermore, to address the lack of support schemes for complex networks in existing accelerators, configuration and scheduling methods such as grouped reuse computation and line-by-line multi-timestep computation are proposed, based on our accelerator architecture. These methods enable acceleration of deep networks with large numbers of channels, feature map sizes, and even for object detection networks.

In summary, the main contributions of this paper are as follows:

1. A clock-driven accelerator architecture that achieves efficient acceleration of SNNs with convolutional topologies.
2. The proposed acceleration structure can be flexibly configured according to the network type, and it can support the current mainstream convolutional networks and the network with residual structures.
3. The proposed methods of grouped reuse computation and line-by-line multi-timestep computation address the challenges of accelerating large networks with deep hierarchies, large numbers of convolutional channels, and large feature map sizes, enabling acceleration for more complex tasks such as object detection.

Experimental results demonstrate the performance of the proposed accelerator architecture, which has achieved a process speed of up to 1, 605 FPS and an energy consumption of only 0.65 mJ per image for SCNNs with a LeNet structure, and for deep residual SCNNs used in object detection, it has achieved a processing speed 2.59 times that of the CPU for the residual modules while the power consumption is only 16.77% of the CPU.

The paper is organized as follows. Section 2 introduces relevant background knowledge on spiking neural networks. The specific design of the SCNN hardware architecture is described in Section 3. Section 4 discusses the accelerator scheduling and configuration strategies for different-sized networks. Then, in Section 5, we evaluated the effectiveness of the proposed SCNN hardware architecture and scheduling configuration strategies based on traditional small networks and large residual networks. Finally, Section 6 summarizes our work.

## 2 Backgrounds of SNNs

### 2.1 Spiking neural networks

There are two main characteristics in the structure of SNNs: one is that the information is expressed by discrete spike sequences with precise timing. The input and output of the network, as well as the information transmitted between network layers, are represented by spike sequences instead of continuous values in traditional ANNs. Secondly, compared to ANNs, the neurons in SNNs provide a more in-depth and detailed simulation of biological neuronal behavior.

#### 2.1.1 Information coding

**Figure 1:**
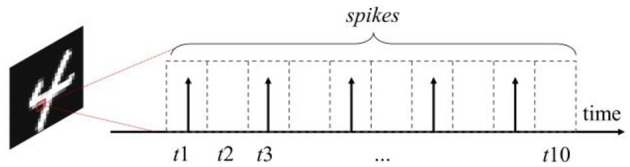
Schematic diagram of poisson coding, where *t*1 − *t*10 denote the 10 time-steps of the spike sequence.

Since the input of SNNs is represented by spike signal, therefore, when applying SNNs to traditional images, each image needs to be encoded into multiple spike sequences of several time steps. And then input the spikes into SNNs in chronological order. The commonly used coding method is rate coding (Gerstner et al., 2014). In this method, the value to be expressed is converted into the average rate of spike generation, i.e., the number of spikes emitted within each sampling time window (Auge et al., 2021). The specific distribution of the spikes within the sampling time window can be done directly by means of equally spaced distribution; it can be also done by means of random distribution according to a probabilistic model, usually using the Poisson distribution model, which is known as Poisson Coding. The schematic of Poisson Coding is shown in Figure 1, taking an example that one pixel value is encoded into a spike sequence of 10 time-steps. In this graph, the emission of spike in each time step from *t*1 to *t*10 follows a Poisson distribution with normalized pixel values as the probability. The energy consumption for information transmission and computation in the network is significantly reduced by using spikes to represent information (Nunes et al., 2022).

As the development of deep SNNs progresses, trainable coding methods have been proposed and rapidly gained widespread application. Trainable coding directly utilizes the first neuron activation layer of the network as a coding layer. The real-valued input images undergo convolutional operations, and then their results are fed into a spiking neuron layer. The activated neurons emit spikes, completing the spike coding of the original image (Wu et al., 2024). For *T* timesteps of the SNNs, the above computation is repeated *T* times. The weights in the coding layer can be trained along with the network, which makes trainable coding show higher precision in deep network structures compared to rate coding. However, under adversarial and interfering conditions, the robustness of trainable coding is still not as good as rate coding (Kim et al., 2022). Additionally, because trainable coding is essentially a real-valued input layer of the network, it requires higher energy consumption to implement and higher costs for hardware deployment.

#### 2.1.2 Neuron model

The input and output of traditional artificial neural network neurons are continuous real values. The neurons perform weighted sum operations on the input signal, and then output the signal through a non-linear activation function (Yamazaki et al., 2022). In SNNs, the behavior of spike neurons is mainly controlled by membrane potential and activation threshold. The spike signal received by the neuron dendrites changes the membrane potential of the neuron. And when the membrane potential accumulated by the neuron reaches the activation threshold, the neuron fires a spike signal from the axon to the neuron at the next layer.

There are several specific neuron models for building SNNs. Take the leaky integrate-and-fire (LIF) model (Gerstner and Kistler, 2002) used in this work as an example. The input signals *X*(*t*) are integrated to the membrane potential *V*; in addition, the membrane potential of LIF neurons also leaks and decreases over time. The mathematical expression of this behavior is shown in Equation 1:


(1) $$\begin{array}{l} \tau \frac{dV}{dt}=V_{reset}-V+X\left(t\right) \end{array}$$

where τ represents the time constant, which is a hyperparameter of the neuron. And when the membrane potential *V* exceeds the threshold voltage *V*_*th*_, the neuron's output will emit a spike (represented by the value 1 for spike emission, 0 for no spike) and reset the membrane potential *V* to the reset voltage *V*_*reset*_.

To facilitate calculation, in the training and inference of the network, the Euler's formula is used to approximate the above continuous differential equation as a discrete difference equation. And the continuous time is also discretized into several moments, here referred to as timesteps. The differenced equation is shown in Equation 2:


(2) $$\begin{array}{l} V\left[t\right]=V\left[t-1\right]+\frac{1}{\tau }\left(X\left[t\right]-V\left[t-1\right]+V_{reset}\right) \end{array}$$

where τ is taken to the power of 2 so that a shift operation can be used instead of division. By using Equation 2, only shifters and adders are needed to calculate the potential of neurons.

### 2.2 Spiking convolutional neural networks

With the rapid development of artificial intelligence in recent years, the ideas of deep networks and convolutional structures in CNNs have been introduced into SNNs. SCNNs retain the convolutional structure of local connection and weight sharing in CNNs, and uses the neurons in SNNs to activate the result of convolution. In SCNNs, binary pixel information is used to represent the presence or absence of spikes. SCNNs make it possible to combine the performance of CNNs with the low power characteristic of SNNs.

**Figure 2:**
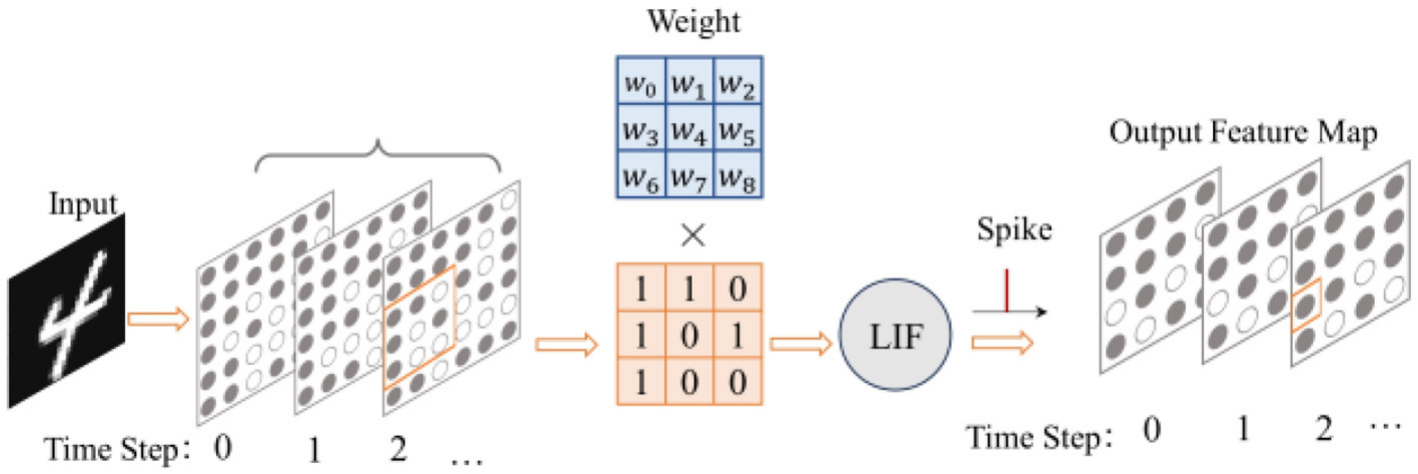
The process of spiking convolution.

Like the convolutional layers in CNNs, the spiking convolutional layers in SNNs play a role in extracting input features spatially, but their input signals are a set of spiking sequences instead of continuous values. Assuming that the total number of timesteps in the network is *T*, the original image needs to be coded into spikes and then enters the spiking convolutional layer as *T* spiking images. Therefore, the convolution operation can be divided into convolving each of the *T* binary spiking images separately, and the result of each calculation is passed to the subsequent LIF neurons, as shown in Figure 2. The mathematical representation of spiking convolution is shown in Equation 3:


(3) $$\begin{array}{l} X\left[x^{\prime },y^{\prime },C_{0},t\right]=\underset{C_{i}}{\sum }\underset{k_{x},k_{y}}{\sum }w\left(k_{x},k_{y},C_{i},C_{0}\right)*s\left(x+k_{x},+y+k_{y},C_{i},t\right) \end{array}$$

where *x*′ and *y*′ are the spatial position coordinate of the calculated output feature map element, *x* and *y* are the corresponding coordinate of the sliding window in the input feature map, *k*_*x*_ and *k*_*y*_ are the coordinate of the convolution kernel element, *C*_*i*_ and *C*_0_ are the input channel and output channel index of the convolutional layer, *w* is the convolution kernel weight, *s* is the input spike, and *X* is the result of the spiking convolution, which is also the input to the LIF neuron. Each element of the output feature map is calculated by the corresponding LIF neuron. The output feature map is also a binary spiking image, and is input to the next layer.

Currently, the training of SCNNs mainly adopts two methods: ANN conversion method and gradient descent method based on surrogate function. Due to the fact that information in SNNs is represented by spike sequences, it is necessary to solve the problem in SNN training that the internal state variables and error functions of SNNs are indifferentiable. For the ANN conversion method, the solution lies in conducting backpropagation in the designed ANN firstly, and then converting the trained ANN into SNN with similar structures. However, this method also has the limitations such as only using the ReLU as the activation function, only using average pooling, not allowing bias and so on. The core idea of the second method is to approximate the indifferentiable spike-neuron activation function in SNN with a similar but smooth and differentiable surrogate function (Neftci et al., 2019; Guo et al., 2023). In recent years, the performance of these two training algorithms has been continuously improved, so that the SCNNs in recent years have achieved the performance comparable to that of CNNs with a similar structure. This has also promoted the development of SNNs toward deeper network structures and higher performance.

For the hardware implementation of SNNs, event driven methods are often used in early work based on MLP structure. This method only updates the neuron state when the spike event arrives, and requires more complex event sequencing and storage logic and neuron state calculation logic (Naveros et al., 2017; Li et al., 2021). And it is a challenge to implement the convolution operation based on the sparse data structure of event flow. In recent years, some studies have strived to solve the problems of high latency and low performance in SNN hardware caused by sparse spike events. Xu et al. (2025) designed an efficient data stream with multi bit weight data movement to improve data sharing for computations spanning across both time and space in the systolic array accelerator. The SATO (Liu et al., 2024) accelerator achieves significant latency and energy reduction by exploiting temporal-unrolled parallelism for cross-timestep membrane potential accumulation and employing a bucket-sort-based dispatcher for balanced workload distribution and input spike reuse across PEs. By the method of algorithm-hardware co-design, STELLAR (Mao et al., 2024) reduces the time step through the few spikes backpropagation (FSBP) training method, and improves the data reuse rate of hardware through the spatiotemporal Row Stationary (stRS) dataflow. Another study, COMPASS (Wang et al., 2024) combined dynamic inferred spike sparsity with in-memory computation to improve the computational efficiency of the SNN accelerator.

Unlike in the event-driven approach, in clock-driven SCNNs, the state of each neuron is computed and updated at each time step according to the input at that time (Wu et al., 2023). It is similar to the mechanism of updating the state of registers at each clock. The input of each time step retains the original image structure, only the pixel values are binarized. So, convolution operations can be implemented easily on such input images. The clock-driven approach reduces the complexity of the hardware implementation, facilitates the implementation of larger networks, and also increases the computational frequency.

## 3 Hardware architecture

### 3.1 Architecture overview

**Figure 3:**
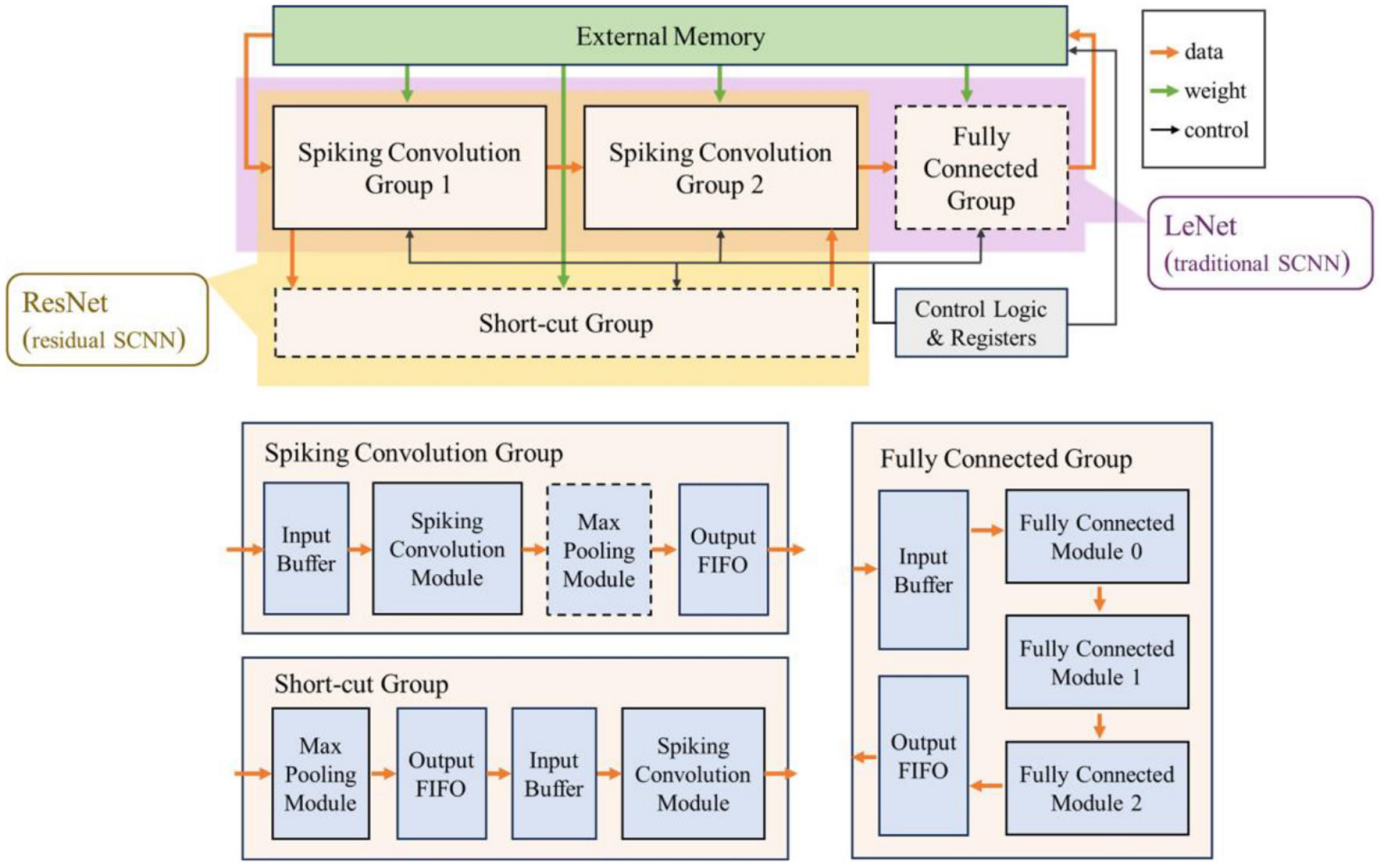
The overall architecture and dataflow of the accelerator.

The overall architecture and dataflow of the accelerator is shown in Figure 3. The accelerator consists of a spiking convolution group, a fully connected group, a short-cut group, external memory, control logic and configuration registers. The parts indicated by dashed lines are optional modules depending on the different network structures. We configure the network based on the widely used LeNet structure and ResNet structure in current research to represent traditional convolution topologies and residual convolution topologies, and build the corresponding hardware accelerator. When configured as LeNet, the accelerator includes a fully connected group (as shown in the purple part of Figure 3); for ResNet structure, a short-cut group is introduced in the accelerator (as shown in the yellow part in Figure 3).

The spiking convolution group includes an input buffer, a spiking convolution module, a max pooling module, and an output FIFO. The max pooling module is also an optional module determined by the network structure. The fully connected group consists of an input buffer, several fully connected modules (taking 3 as an example), and an output FIFO. The short-cut group includes an optional max pooling module, an output FIFO, an input buffer, and a spiking convolution module. Note that the input of the short-cut group comes from the input buffer in the first spiking convolution group, and the output data will be transmitted to the spiking convolution module in the last spiking convolution combination to superimpose the calculation results of the shortcut connection.

The external memory stores the input data, weight parameters, and output data of the accelerator. The data transmission path and weight transmission path are indicated by the orange and green arrows in Figure 3, respectively. The control logic and registers are used to control the operation of each module. Meanwhile, the accelerator has a certain degree of programmability, and the partial specifications of the network can be adjusted online by configuring the registers through the external interface.

### 3.2 Input buffer module and output FIFO module

**Figure 4:**
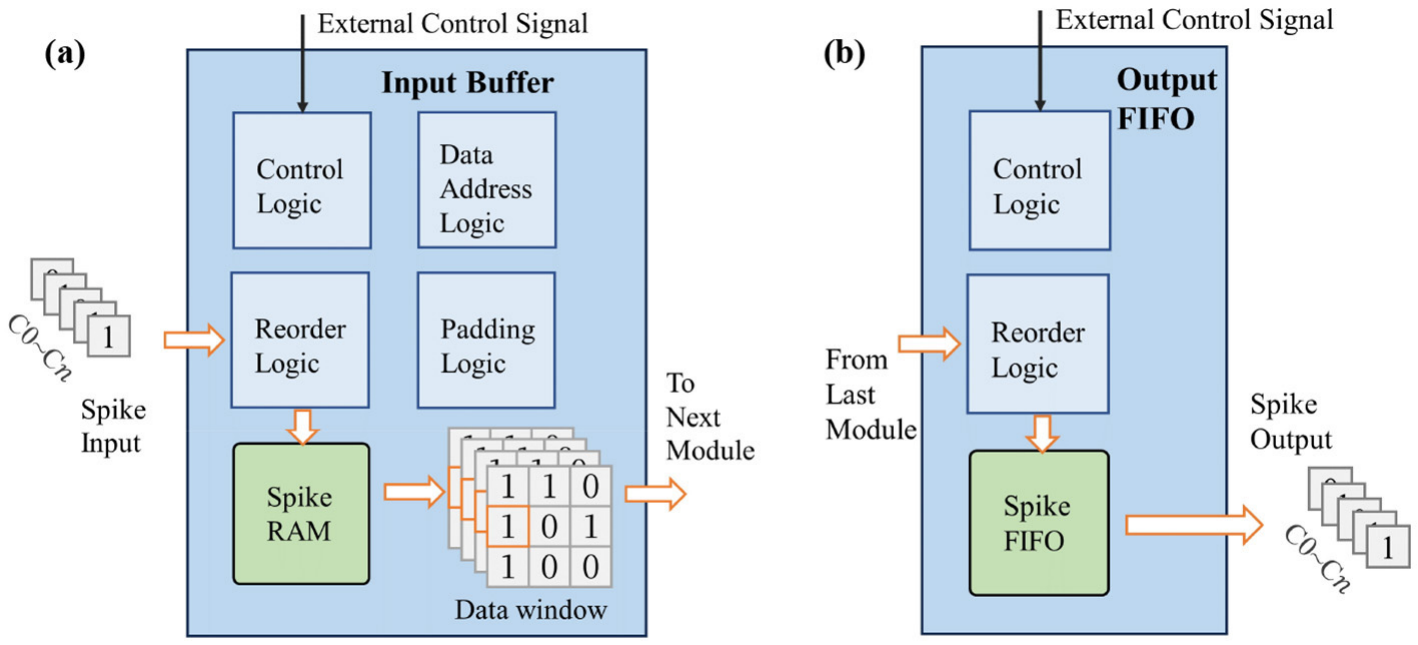
The design of input buffer and output FIFO modules.

The input buffer module is mainly used to receive the input feature map data and reorder it into the sliding window required for convolution. Its design is shown in Figure 4a, where *n* represents the number of data channels. It includes spike RAM, a data window, control logic, as well as logic for address calculation, data reordering, and padding functions. The input buffer module receives data from the external memory or the previous stage's output FIFO. Due to the binary nature of the spikes, data can be transmitted preferentially according to the channel dimension. Therefore, the input feature map is traversed in the order of [*C*,*X*, *Y*, *T*] (where *C* denotes the input channel dimension, *X* denotes the width dimension, *Y* denotes the height dimension, and *T* denotes the timestep dimension) and stored in the spike RAM of the input buffer. When the stored data is sufficient to start subsequent calculations, the spike data is read out from the spike RAM and rearranged according to the configured network structure, and subsequently stored in the data window for next modules. When the subsequent module is a convolution or pooling module, the data window is configured as a two-dimensional register set with the same size as the computation kernel; when the subsequent module is a fully connected module, it is configured as a one-dimensional input spike queue; the padding operation is also completed during the data loading process within the input buffer module; the address calculation logic is used to generate the read and write addresses of the spike RAM. The control logic is used to receive configuration information, enable signals, and other signals transmitted by external control signals, and control other internal sub-modules to operate in the above-mentioned manner. All other modules also have similar control logic, which will not be repeated in the following content.

The output FIFO is used to cache the calculation results of the previous module. It includes reorder logic, spike FIFO, and control logic. When a line of data is cached, it transfers the data to the next stage's input buffer or external memory, as shown in Figure 4b. The order of data transmission is consistent with that of the input buffer module, and the output feature map is stored in the order of [*C*,*W*,*H*,*T*]. Since only one line of data needs to be temporarily stored, a FIFO structure can be directly used for caching, enabling synchronous receiving and sending of data.

### 3.3 Clock-driven spiking convolution module

**Figure 5:**
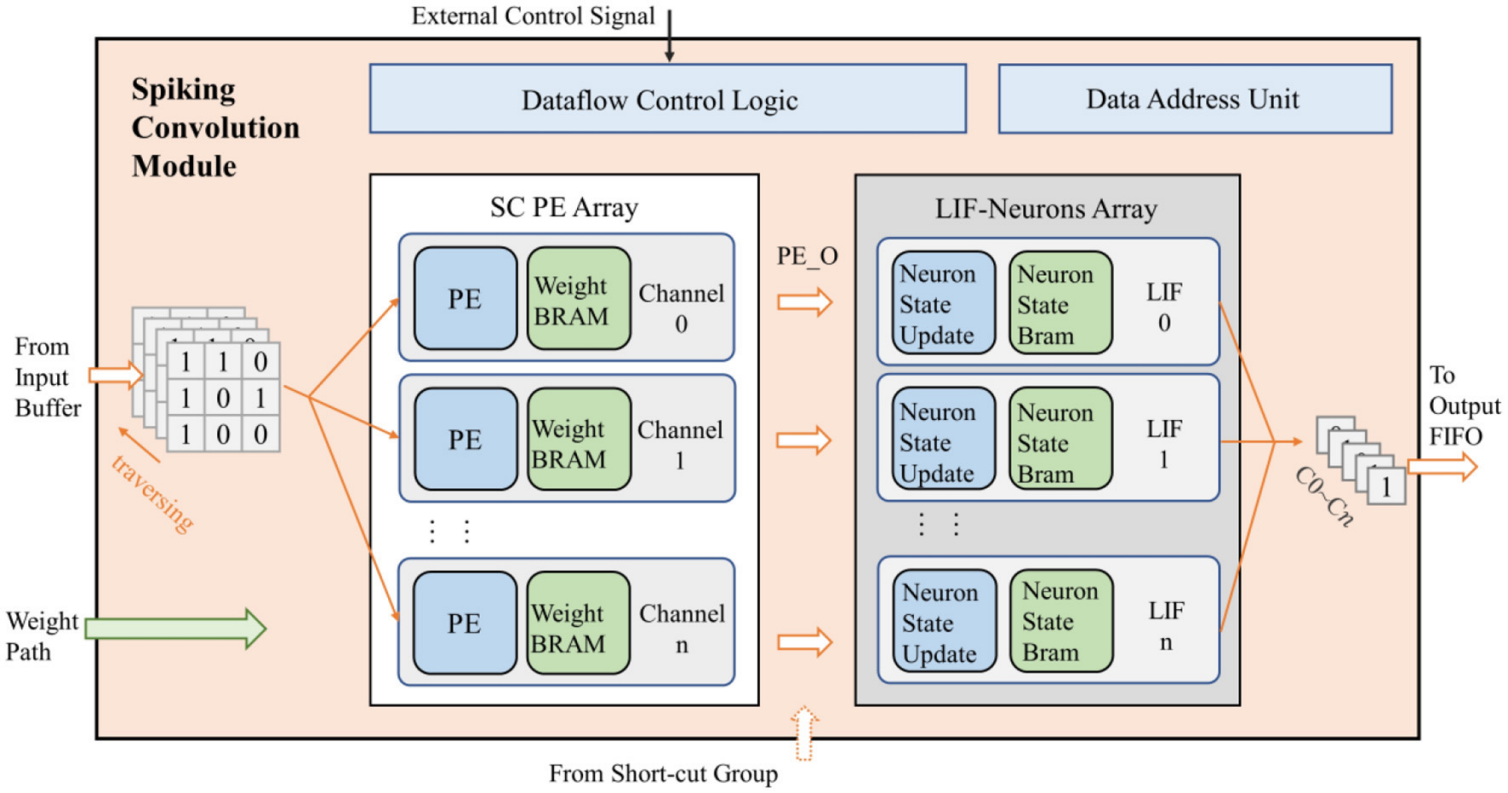
The design of spiking convolution module.

The spiking convolution module mainly includes a spiking convolution (SC) processing element (PE) array, an LIF neuron array, data flow control unit and data address calculation unit, as shown in Figure 5. Specifically, when configured as a residual structure, the spiking convolution module of the short-cut group does not include the LIF neuron array. The convolution module adopts a parallel structure according to the output channel to speed up the computation. It contains *n* independent computing threads (*n* is the number of output channels). Each computing thread consists of a convolution PE, an LIF neuron activator, a weight BRAM, and a neuron-state BRAM.

The input of the spiking convolution module includes a data input path connected to the data window of input buffer, a path for receiving weight data transmitted from off-chip memory, and a path for control signals from the accelerator's control logic and configuration registers. Besides, there is also a data output path in this module that transmits the computational results to the output FIFO. The general operating mechanism of the entire module is described as follows.

Firstly, the control signals convey the specifications of the network (such as the number of channels, feature map sizes, etc.) into the spiking convolution module. Subsequently the weights of the network are passed in from the weight path and stored in the weight BRAM within the module. Then the input buffer fills the input feature map into the data window, the spiking convolution module reads the data in the data window by input channel and broadcasts it to each computational thread. The convolution PE retrieves the weights at the corresponding positions and performs convolution calculations based on the incoming data. After the data of all input channels are passed in, PE gets the final computation result PE_O, which is output to the neuron unit to perform the activation operation. The neuron calculates whether to fire a spike based on the input. The activation results from all channels are then output through the data output path. For residual structures, a data input path for the computation result of short-cut group is added in the last convolution module of the residual path. The PE_O calculated by this module needs to be superimposed with the computation result from the short-cut group before being input into the LIF neuron for activation.

**Figure 6:**
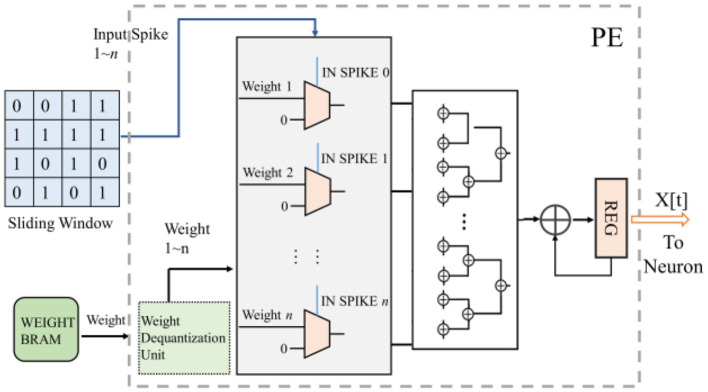
The computation process of the convolution kernel of SCNNs, where *n* is the amount of data in the sliding window used in one computation. The configuration of this work is to calculate the data in one row of the sliding window in one clock cycle, and that means for an m*n convolution kernel, it takes m clock cycles to complete the convolution operation of one sliding window.

For the convolution operation in PE, since the input of SCNN's each layer is a binarized spiking image, the multiplicative accumulation operation of convolution can be transformed into the accumulation operation of weights. Therefore, multiplication operations are no longer required in the convolution process of SCNNs, which simplifies the circuit and greatly reduces the power consumption. The computational process is illustrated in Figure 6. The spiking convolution module reads the weights of the current layer from external memory and caches them in on-chip BRAM. The corresponding weights in the BRAM are read into the accumulator operand register based on the coordinates of the input data. The weights are represented as 8-bit fixed-point numbers or quantized to 8-bit integers (in this case, the read-out weights need to be converted to fixed-point form through a de-quantization unit). The values in the data window are used as the gating signals for the corresponding weight, to choose whether the operand entering the adder tree is the weight or 0. Subsequently, the adder tree calculates the accumulated result, which is stored in the accumulation result register until the final computation result PE_O is generated.

**Figure 7:**
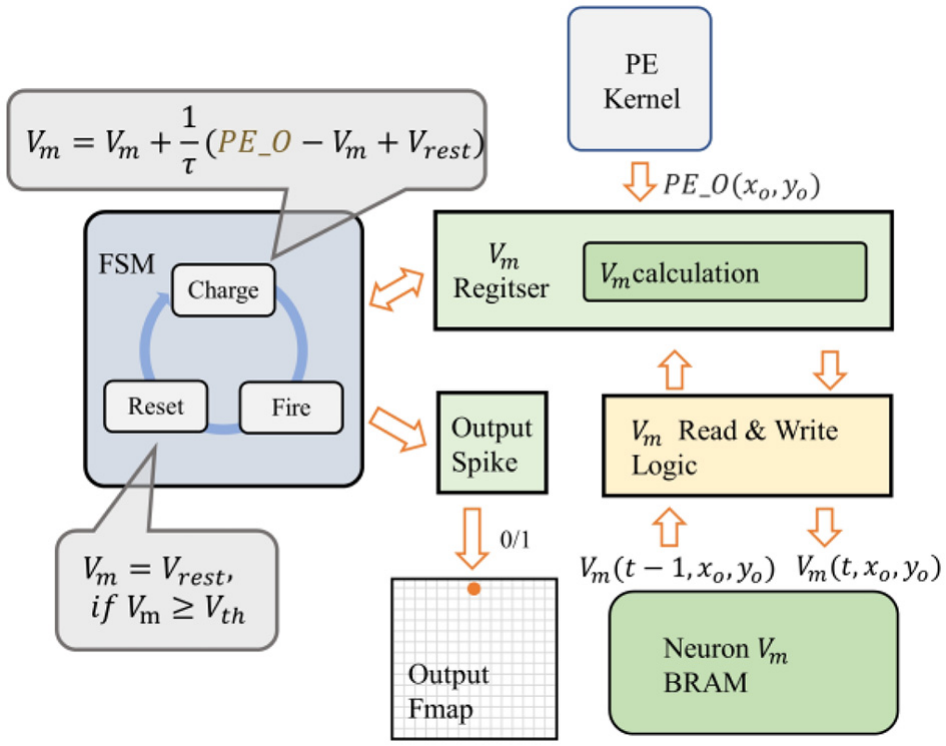
The reuse strategy for the neuron operation logic.

In the output feature map of SCNNs, each pixel's value is obtained by the activation of a corresponding neuron. To reduce the resource occupied by neurons, we designed a reuse strategy for the neuron operation logic in the neuron module of the convolutional layer, as shown in Figure 7. Only one neuron-state update module is configured for each computational thread. When PE_O arrives, the membrane potential value of the neuron at the current output feature map's coordinate is read from the neuron-state BRAM to the neuron-state update module to calculate its new membrane potential. If the membrane potential exceeds the threshold, the neuron fires a spike and resets the membrane potential. Use 1 or 0 to indicate whether the neuron is activated or not, and the result of the activation is written to the corresponding location in the output line buffer. At the same time, the neuron-state update module saves the current membrane potential value back into BRAM.

After calculating one pixel's value of the output feature map, the data window slides a step to calculate the pixel's value at the new coordinate. The above process is repeated until the calculation of all input feature maps in the buffer is completed. That also means the data processing of the t time-step is completed, and the input buffer will load the spiking image of the next time-step and start the processing of *t*+1 time-step.

Data flow control unit is mainly responsible for controlling the data transfer between the components. While the data address calculation unit is responsible for generating the coordinates of the sliding window and the coordinates where the activation results are written to output line buffer. And it is also responsible for providing the retrieval address of weight BRAM and neuron state BRAM.

### 3.4 Max pooling and fully connected modules

**Figure 8:**
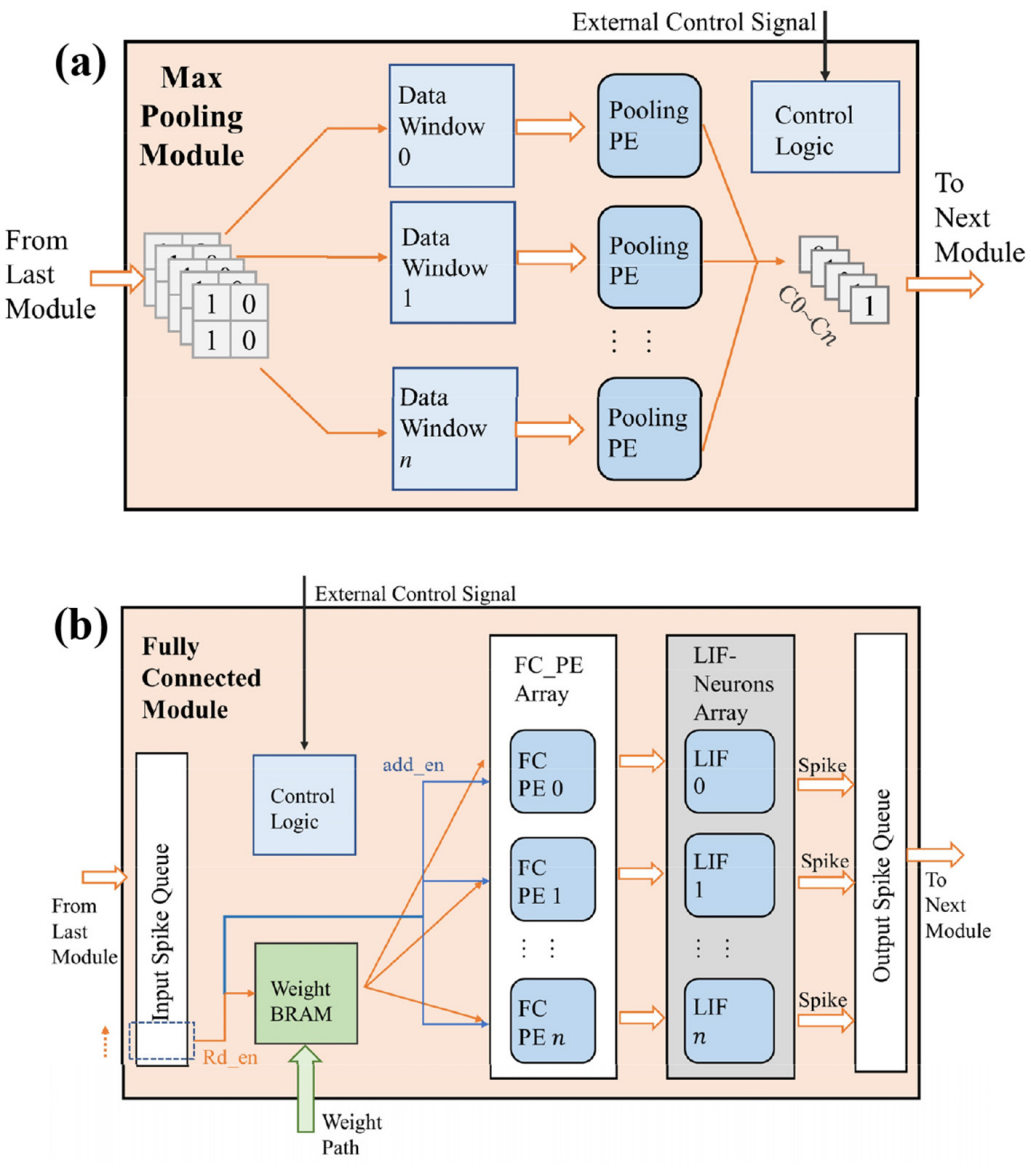
The design of max pooling and fully connected modules.

The max pooling module consists of a data window, pooling PE, and control logic, as shown in Figure 8a. Depending on the location of the max pooling module, its input path differs. For the max pooling module in the residual structure, its input comes from the bottom-right 2 × 2 portion of the data window in the input buffer. For the max pooling module following the spiking convolution module in the LeNet structure, the convolutional results are temporarily stored in a line buffer and then input into the max pooling module.

Since the output of spiking neurons is represented by spikes, if we use the average pooling, the spikes will be reconverted to floating point numbers, which is inconsistent with the design idea of SCNNs. So, we use max pooling. Max pooling of SCNNs is easy for hardware implementation because it only needs each PE performs a bitwise OR operation on the data stored in the data window. The pooling layer continues the design of parallel computing threads in the convolutional layer. Each computing thread completes the pooling operation of one output channel, and finally generates a complete output feature map for the next layer's components.

Figure 8b shows the architecture design of the fully connected (FC) module. It consists of an input spike queue, weight BRAM, a FC PE array, a LIF neuron array, an output spike queue, and control logic. Similar to the design of convolutional layers, a FC module containing *n* neurons can be considered as a convolutional layer with *n* output channels, but the output feature map's size of each output channel is only one. Therefore, in the FC module, the membrane potentials of neurons only need to be stored in their registers, and there is no need to store the membrane potential of different locations to the corresponding address of BRAM, as what we do in the convolution layer. For the input feature map, it is spread into a one-dimensional spike sequence stored in the input spike queue. The FC module sequentially reads the pixel values in the input buffer, and then takes them as the read enable of the weight BRAM and the gating signals of the accumulation operation for the PE on each output channel. Until all the pixels in the input buffer are read, each PE passes the cumulative value of the weight to the neuron array for activation to get the feature map stored in output spike queue under at the current time step. Although each pixel of the input feature map needs to be retrieved serially in order to obtain the output result of one timestep, the processing time of the fully connected module is still short due to the simple computational logic of FC module and the low data volume after two pooling's dimensionality reduction.

## 4 Configuration and scheduling methods of accelerator

### 4.1 Timestep pipeline computation for small-scale SCNNs

**Figure 9:**
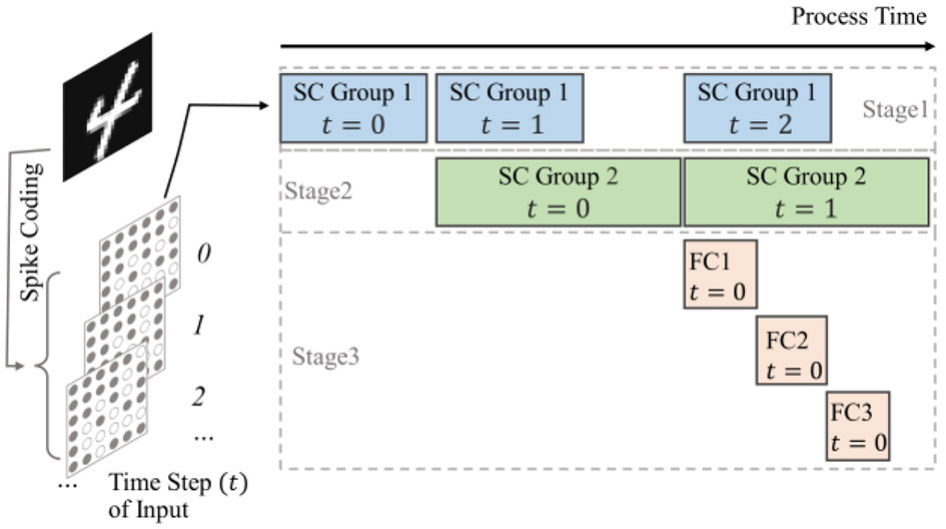
Schematic of timestep pipeline computation for small-scale SCNNs, where the blocks in each stage indicate which module is working and the data from which time step is processed.

For small networks, it is possible to directly implement all network layers on-chip and store the weights of each layer as well as the inputs of the network directly in the on-chip BRAM as well. Each computational module is specially designed based on the specifications of the corresponding network layer, and there will be no idle computational units, thus the utilization efficiency of hardware resources can be improved. Since all modules are implemented on-chip, these modules can be scheduled in a pipeline to accelerate computation. Taking the LeNet structure as an example, as shown in Figure 9. The network is structured as a three-stage pipeline: the spiking convolution group 1 constitutes the first stage of the pipeline. The spiking convolution group 2 constitutes the second stage of the pipeline, and the full connection group comprises the remaining part of the pipeline. Between each pipeline stage, input buffers and output FIFOs are used for temporary data storage. Taking the first stage as an example, after it finishes processing the input data of *t* time-step, it passes the processed data to the next stage and starts processing the input of the *t*+1 time-step if the next level of the pipeline is free; if the next stage is busy, it keeps waiting. With such scheduling, each network layer can keep working after the pipeline is filled up, so that the hardware resources can be used efficiently and the processing performance of the hardware can be improved.

For residual structures, the short-cut group shares the input buffer with the spike convolution group 1, and the output data is superimposed into the spike convolution group 2. Therefore, the max pooling module in the short-cut group is in the first stage of the pipeline together with the spike convolution group 1, while the spiking convolution module in the short-cut group is in the second stage. Similarly, the input buffers and output FIFOs of short-cut group are used to cache data between these two pipeline stages. To ensure that the computational results of the two parallel paths align in the output feature map, both paths maintain consistent coordinate of sliding window at each stage.

### 4.2 Layer-by-layer reconfigurable computation for deep SCNNs

**Figure 10:**
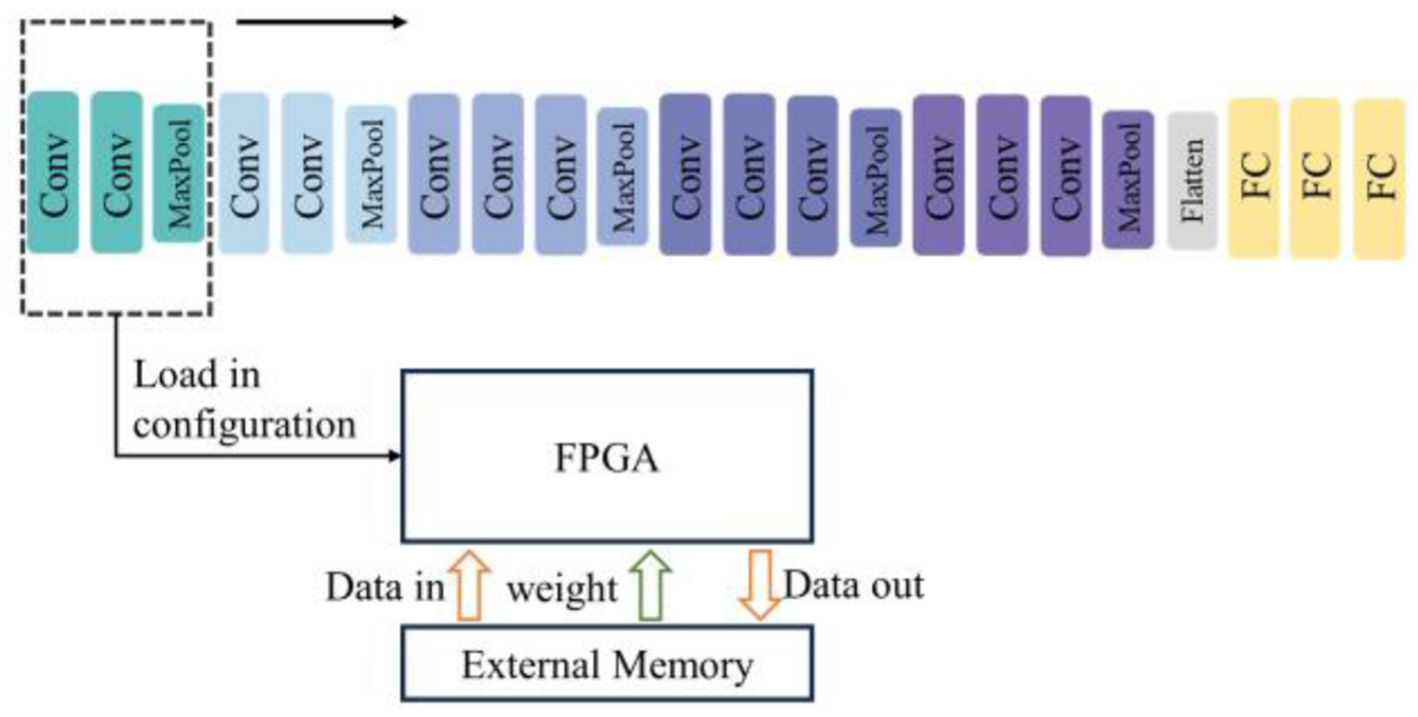
Schematic of layer-by-layer reconfigurable computation for Deep SCNNs.

As the number of network layers increases, limited hardware resources may not be sufficient to implement the entire network. In such cases, only some layers of the network can be implemented in hardware, and the whole computation is completed layer by layer through reconfiguration and reuse strategies, as shown in Figure 10. This means that the computational modules in the hardware need to support specifications such as the number of channels and feature map sizes for each network layer. The specification information of the layer being accelerated currently is transmitted through the external interface, and then the corresponding computational resources in the accelerator are activated. The weights of the entire network need to be stored in external memory first, and only the weights of the layer being accelerated currently are read from DDR and stored in BRAM.

### 4.3 Grouped reuse computation for wide SCNNs

Deep neural networks not only have lots of layers but also include many wide convolutional layers with a large number of channels. When applying the layer-by-layer reconfigurable computation strategy discussed in the previous section, excessive convolutional channels can still prevent a single layer from being fully implemented in hardware. To address this issue, we propose a scheduling strategy for convolutional operations based on grouped loop unrolling. By grouped reusing computational resources within each layer's module, wide convolutional layers can operate within hardware with limited resources.

**Figure 11:**
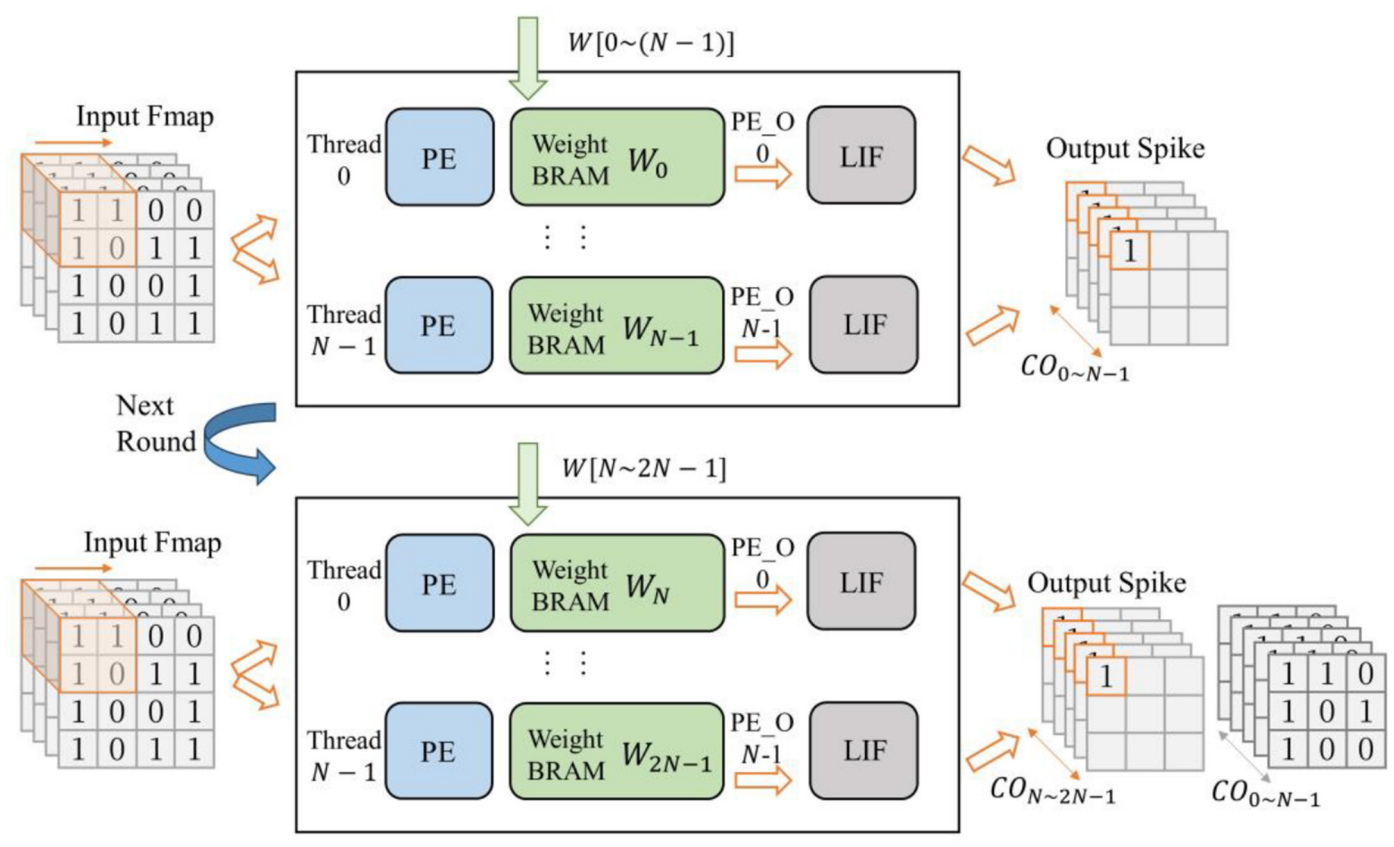
Schematic of grouped reuse computation for wide SCNNs (first-stage of convolution), where $\frac{N_{CO}}{N}=2$ is used as an example.

In order to obtain usable computational results as quickly as possible, the accelerator's first-stage input buffer will cache all the input channels of the input feature map coming from the external memory. Subsequently, the first-stage spiking convolution layer, based on the number of parallel computing threads *N* in the hardware, divides the weights and the output feature map into $\frac{N_{CO}}{N}$ groups on the output channel dimension (assuming the number of output channels is *N*_*CO*_).Then, the first group of weights is loaded, and convolution calculation is performed by the method of loop unrolling to obtain the result for the output feature map on channel *C*_0~*N*−1_. After that, the data window of the input buffer returns to the starting point to reload the data. And the weights for the next group of output channels are read from the DDR into each weight BRAM, to complete the convolution calculation for the output feature map on channel *C*_*N*~2*N*−1_. The process is illustrated in Figure 11. This continues until the computation for all channels of the output feature map is completed.

The accelerator incorporates two-stage pipelined spiking convolutional layers. For the second-stage spiking convolutional layer, in addition to the output channel based grouped reuse described previously, it also needs to perform grouped calculations in the input channel dimension. This is because the output from the previous layer is generated in accordance with the grouping of output channels. If the second convolutional layer waits for the entire input feature map to be generated before starting its calculations, it would result in prolonged pipeline stalls and reduced hardware resource utilization efficiency.

**Figure 12:**
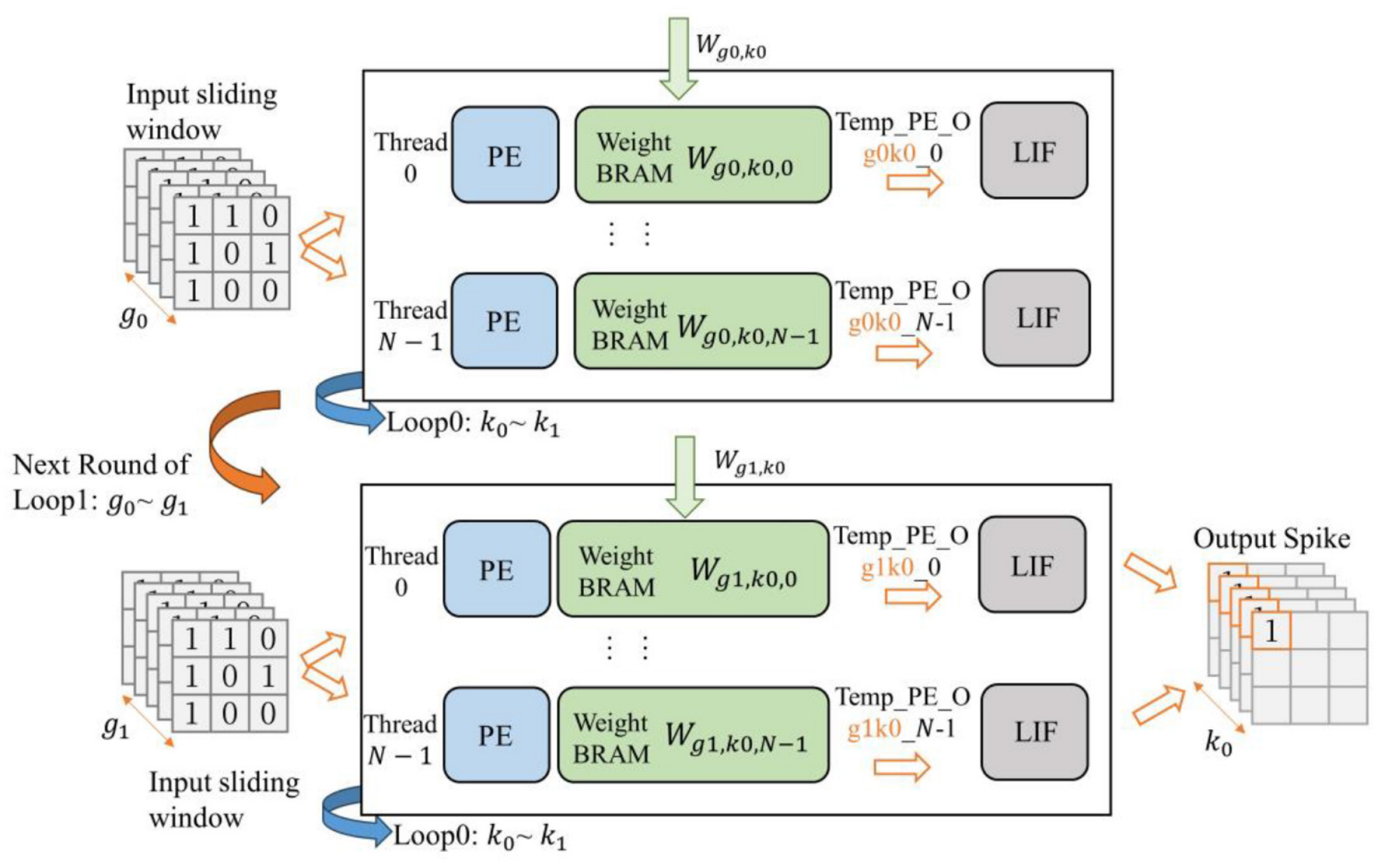
Schematic of grouped reuse computation for wide SCNNs (second-stage of convolution), where $\frac{N_{CI}}{N}=\frac{N_{CO}}{N}=2$ is used as an example.

For the second convolutional layer, its input data can be viewed as divided into $\frac{N_{CI}}{N}$ groups along the input channel dimension (assuming the number of input channels is *N*_*CI*_). Meanwhile, the weights are also further grouped based on the input channels on the basis of previously grouping by the output channels, resulting in a total of $\frac{N_{CI}}{N}\times \frac{N_{CO}}{N}$ groups. To facilitate explanation, $g_{i}\left(i=0,1,\ldots ,\frac{N_{CI}}{N}-1\right)$ is used to represent each group of input feature maps grouped by input channels, and $k_{j}\left(j=0,1,\ldots ,\frac{N_{CO}}{N}-1\right)$ is used to represent each group of output feature maps grouped by output channels. When the input feature map *g*_0_ is loaded, the spiking convolutional layer also loads the corresponding weights $W_{g_{0},k_{j}}\left(j=0,1,\ldots ,\frac{N_{CO}}{N}-1\right)$ for *g*_0_. Once *g*_0_ is ready, the spiking convolutional layer can proceed with the previously described output channel based grouped method, performing loop unrolling calculations within the input channel range of *g*_0_. Each computation thread produces $k_{\frac{N_{CO}}{N}-1}$ sets of intermediate results (called *Temp*_*PE*_*O*_*g*_0_). Similarly, this process is repeated until all of *g*_*i*_ are completed, and the complete output feature map, namely $k_{0}\sim k_{\frac{N_{CO}}{N}-1}$, can be obtained. This entire process is illustrated in Figure 12.

According to the spiking convolutional computation process described by Equation 3, when the input feature map is not grouped, the convolution result *PE*_*O*_*f*_(*f* = 0, 1, …, *N*−1) of each computing thread corresponds to the spiking convolutional computation result *X*[*x*′, *y*′, *jf, t*] for the current sliding window position and timestep; However, when performing convolution calculations on the grouped input *g*_*i*_, the result *Temp*_*PE*_*O*_*f*,_*g*__*i*__ is only a part of *X*, denoted as $X_{i}^{\prime }\left[x^{\prime },y^{\prime },jf,t\right]$. And their relationship is expressed in Equation 4:


(4) $$\begin{array}{l} X\left[x^{\prime },y^{\prime },jf,t\right]=\underset{i}{\sum }X_{i}^{\prime }\left[x^{\prime },y^{\prime },jf,t\right] \end{array}$$

From the above equation, we can see that the intermediate calculation result *Temp*_*PE*_*O*_*f*,_*g*__*i*__ from PE cannot be directly used for the activation result of LIF neuron. It needs to be temporarily stored until the calculation result for the last group of input feature maps $g_{\frac{N_{CI}}{N}-1}$ is generated. To achieve this, we utilize the accumulation relationship in Equation 4 and accumulate the intermediate calculation results into the membrane potential value of the neuron. Therefore, under the grouped reuse computation method for wide SCNNs, the integrating equation for the LIF neuron described in Equation 2 needs to be modified as shown in Algorithm 1.

**Algorithm 1:** Convolutional intermediate result staging algorithm based on membrane potentials.

**Input:** the convolution results *X*′[*i, t*] under grouped reuse, the time step *T*, group number $\frac{N_{CI}}{N}-1$ divided by input channels.
1: **for** *t* = 0 → *T*−1 **do**
2: **if** *t* = 0 **then**
3: *V*[*t*−1] = 0
4: **end if**
5: **for** $i=0\to \frac{N_{CI}}{N}-1$ **do**
6: **if** *i* = 0 **then**
7: $H\left[t,i\right]=\left(1-\frac{1}{\tau }\right)V\left[t-1\right]+\frac{1}{\tau }\left(pe\text{\_}o\left[t,i\right]+V_{reset}\right)$
8: **else**
9: $H\left[t,i\right]=H\left[t,i-1\right]+\frac{1}{\tau }pe\text{\_}o\left[t,i\right]$
10: **end if**
11: **end for**
12: **end for**
**Output:** membrane potential *H*[*t*] at the current timestep after integrating is completed 2

When each group of convolutional results is fed to the LIF neuron, the new membrane potential is calculated according to the above algorithm, thereby achieving the effect of temporarily storing the intermediate results of the convolution. The leakage term of the LIF neuron is only calculated when the first-group convolution result *X*_0_ arrives, and is not calculated for other intermediate results. The activation results are calculated after the last group of results arrives.

### 4.4 Line-by-line multi-timestep computation for large-size feature maps

Neural networks under complex tasks, in addition to the deeper and wider network structures, there is also the characteristic of larger feature map sizes, such as tasks like ImageNet (Deng et al., 2009) image recognition and object detection. At this time, storing a complete feature map on-chip would also consume more resources. For SNNs, it is also necessary to consider the issue of saving the state of neurons at each timestep. The spiking convolution module in our accelerator is designed with dedicated RAM blocks to store the membrane potential of each neuron corresponding to each pixel position in the output feature map from the last timestep. These old neuron state values are used to compute the activation result for that position at the current timestep. When the size of the output feature map is large, the number of neurons that need to be stored also increases, which will require more RAM resources. To address this issue, a scheduling strategy of line-by-line multi-timestep computation is proposed in our work to enable networks with large feature map sizes to be implemented on the accelerator and to optimized the resource utilization efficiency.

**Figure 13:**
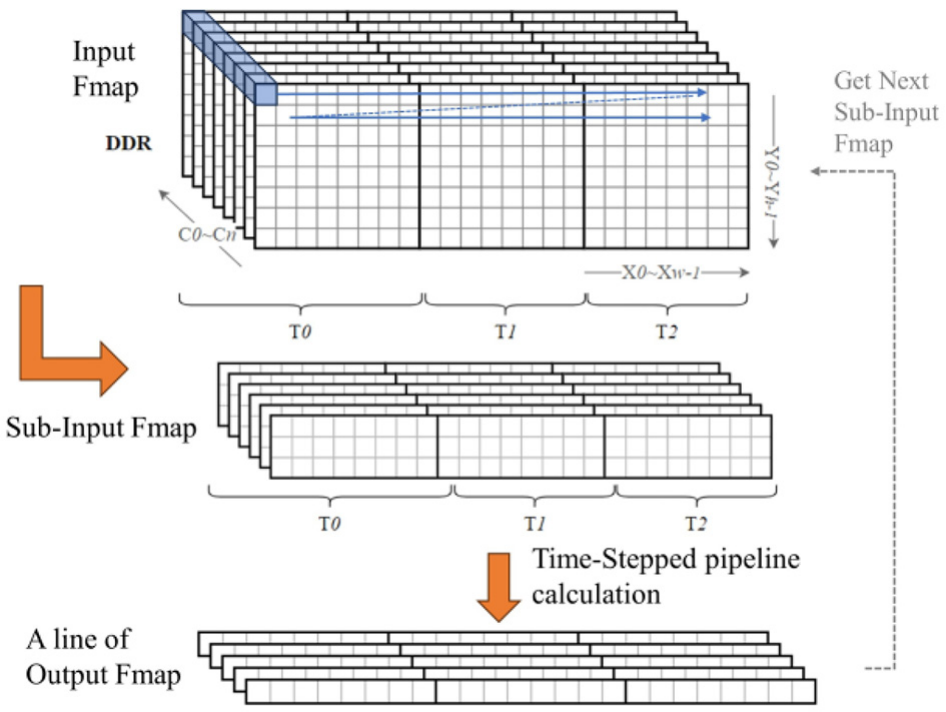
Schematic of line-by-line multi-timestep computation.

The core idea of this method is shown in Figure 13. First, the feature map in DDR is decomposed line by line into several sub-feature maps of *K* rows(*K* is the size of the convolutional kernel); the input buffer only needs to cache the data of one sub-feature map, eliminating the need to store the entire feature map. Then, following the timestep pipeline method designed in the previous context, the first line of the output feature map is calculated; subsequently, the remaining parts of the output feature map are calculated line by line in the same manner. Additionally, there are (*K*−*S*)(*S* is the stride of convolution) lines of overlapping data between every two sub-feature maps; the BRAM can reuse the repeated data through internal reordering logic during the data input process, so there is no need to load the overlapping data when loading a new sub-feature map. Each line of results is immediately output to the input buffer of the next layer, and once the pipeline of the next stage is filled, each line of input data can yield a new line of results.

For the neuron states in SNNs, under this strategy, the neuron state BRAM only needs to store the membrane potential values corresponding to one line of pixels in the output feature map, reducing the storage resource requirements for the neuron states. In this strategy, the input buffer needs to cache the data of the sub-feature map at all time steps; while the convolution module needs to complete the calculation of one line of the output feature map at all time steps, so as to clear the neuron state BRAM and use it entirely for the next line's calculation. Therefore, for the entire output feature map, the data is generated in the order of [*C, X, T, Y*], so this method is called the line-by-line multi-timestep computation strategy.

To further clarify the applicability proposed in this section, Table 1 summarizes their target scenarios, providing a concise reference for readers to understand when and how each strategy is deployed.

**Table 1:** Scheduling strategies and their applicable scenarios.

**Scheduling strategies**	**Applicable scenarios**
Timestep pipeline computation	Small-scale SCNNs
Layer-by-layer reconfigurable computation	Deep SCNNs
Grouped reuse computation	Wide SCNNs with large number of channels
Line-by-line multi-timestep	SCNN layers with large feature maps

## 5 Experiment and evaluation

The experiments in this paper are divided into two parts: the accelerator testing and validation for small traditional convolutional networks and for large residual networks. The former configures the accelerator as a network with the LeNet structure to verify the performance and energy efficiency of the accelerator module architecture, as well as its applicability to basic networks like small traditional convolutional networks. The latter configures the accelerator as residual blocks within large residual networks to validate the applicability of the accelerator's configuration and scheduling strategy to complex networks.

### 5.1 Testing and validation for small traditional convolutional networks

#### 5.1.1 Experimental setup

In this work, we take the LeNet model (Lecun et al., 1998) as the prototype and configure two different scale networks, defined as LeNet-Small and LeNet-Large. Their specific parameter configurations are shown in Table 2. We input the encoded 28 × 28-size spike images into the network according to the time step order. The 10 neurons in the output layer are used to represent the classification result. After processing the input of all the time steps, the category represented by the neuron, which has the highest cumulative number of output pulses in the output layer, is considered as the classification result.

**Table 2:** Configuration of the LeNet models.

**Model**	Configuration^*^
LeNet-small	Input (28 × 28)-6C5-P2-16C5-P2-F120-F84-Output (10)
LeNet-large	Input (28 × 28)-64C5-P2-64C5-P2-F120-Output (10)

* *nCs* indicates that the layer is a convolutional layer containing *n* convolutional kernels of size *s*×*s*; *Ps* indicates that the layer is a max pooling layer with *s*×*s* size; *Fs* indicates that the layer is a fully connected layer containing *s* spiking neurons.

In this work, we wrote the RTL code for the above hardware architecture in System Verilog and implemented the two scales of network mentioned before: LeNet-Small, LeNet-Large, using Xilinx Zynq xz7z035 FPGA board. We used the Spiking Jelly (Fang et al., 2023), an SNN framework, to train the network on the host computer side. In this part, we used the gradient descent method based on surrogate function to train SNNs directly, rather than the ANN conversion method used in most work. So that we can avoid the limitation that max pooling cannot be used in the conversion method. In this method, during forward propagation, the activation is represented by the step function *Heaviside*(*x*), and during backward propagation, the derivative of the sigmoid function *g*′(*x*) is used as the surrogate gradient function. The mathematical expressions for the sigmoid function *g*(*x*) and its derivative *g*′(*x*) are Equations 5, 6:


(5) $$\begin{array}{l} g\left(x\right)=\text{sigmoid}\left(\alpha \left(x-V_{th}\right)\right)=\frac{1}{1+e^{-\alpha \left(x-V_{th}\right)}} \end{array}$$

(6) $$g^{\prime }(x)=\alpha (1-sigmoid(\alpha (x-V_{th}))sigmoid(\alpha (x-V_{th}))$$

where the parameter α = 4.0, such that the maximum value of their gradient is 1.

For the datasets, we used the MNIST dataset (Lecun et al., 1998) and the more complex version, Fashion-MNIST dataset (Xiao et al., 2017), to train and test our proposed architecture in this section. This work uses Poisson encoding to convert each image of these datasets into 10 time-step spiking images and input them into the network.

#### 5.1.2 Evaluation of experimental results

Table 3 summarizes the performance and power consumption of the proposed SCNN accelerator architecture in this paper and compares it with other recent work on SNN/SCNN accelerators. The Intel Loihi and Darwin in the table are ASIC-based SNN hardware accelerators, and the remaining Platforms are FPGA-based SNN hardware accelerators. It can be seen that thanks to the parallel and pipeline strategies adopted in this paper, the LeNet-Small network achieved a recognition speed of 1, 605 FPS at 100 Mhz clock frequency, while also achieving accuracy of 99.1% and 84.6% in the MNIST and Fashion-MNIST, respectively. Its power consumption was only 1.05 W. Compared with the work of the same clock frequency (Zhang et al., 2021; Ye et al., 2023), our LeNet-Small achieves higher recognition speed; although the recognition speed of Fang et al. (2020) is faster, our power consumption is only 23.56% of it, achieving the lowest energy consumption on single image processing in the table.

**Table 3:** Performance and energy consumption compared with other SNN/SCNN accelerators.

**Work**	**Hardware platform**	**Clock frequency /MHz**	**Accuracy on MNIST / %**	**Accuracy on F-MNIST / %**	**Frame rate /FPS**	**Power /W**	**Energy/ (mJ/pic)**
**This work LeNet-small**	**Zynq7035**	**100**	**99.1**	**84.7**	**1,605**	**1.05**	**0.65**
**This work LeNet-large**	**Zynq7035**	**100**	**99.26**	**88.2**	**632**	**1.82**	**2.88**
(Ju et al. 2020)	Zynq ZCU102	150	98.94	/	164	4.6	28
(Fang et al. 2020)	Zynq XCZU9EG	125	99.2	/	2,124	4.5	2.12
	Intel Loihi (ASIC)	/	98	/	671	3.77	5.62
(Zhang et al. 2021)	Zynq 7045	100	97.3	83.3	1,250	1.24	1.0
(Ye et al. 2023)	Kintex 7 XC7K325T	100	99.1	90.3	826	0.98	1.19
(Ma et al. 2017)	Darwin (ASIC)	25	93.8	/	6.25	0.02	3

The bold values in this table are only used to highlight the experimental data corresponding to the work of this paper, with no other special meanings.

For the LeNet-Large model, its accuracy in the MNIST is improved to 99.26%, and the accuracy for Fashion-MNIST is significantly improved to 88.2%; although this is at the expense of part of recognition speed, its recognition speed still maintains 632 FPS, and the power consumption is only increased to 1.82 W. The energy consumption for single image processing in LeNet-Large is still better than that of works such as Ju et al. (2020) and Ma et al. (2017). Compared with other FPGA hardware and neuromorphic chips for SNNs/SCNNs, the SCNN hardware architecture proposed in this paper achieves competitive recognition rate and recognition speed as well as energy consumption performance.

Table 4 summarizes the FPGA resource consumptions of the proposed SCNN accelerator architecture in this paper and compares it with other recent work on SNN/SCNN accelerators. It can be seen that the architecture proposed in this paper has excellent performance, while its hardware resource consumption is significantly better than other FPGA implementation schemes. Even for the larger network, LeNet-Large, our resource consumption such as registers still has a significant advantage over those with similar quantization bit width and network size, such as the work of Ju et al. (2020). In particular, our hardware design does not need to use the DSP resources in FPGA as other high-speed solutions like Zhang et al. (2021) and Fang et al. (2020) do. The proposed SCNN hardware design facilitates the deployment of SCNNs in low-cost FPGA and provides more space for integrating other applications.

**Table 4:** FPGA resource consumptions compared with other SNN/SCNN accelerators.

**Work**	Model configurations^*^	**Bit width**	**LUT**	**Register**	**BRAM**	**DSP**
**This work LeNet-small**	**6C5-P2-16C5-P2-F120-F84-F10**	**8 bit**	**40,703**	**15,365**	**60.5**	**/**
**This work LeNet-large**	**48C5-P2-64C5-P2-F120-F10**	**8 bit**	**81,970**	**35,565**	**239.5**	**/**
(Ju et al. 2020)	64C5-P2-64C5-P2-F128-F10	8 bit	107,273	67,278	264.5	/
(Fang et al. 2020)	32C3-P2-32C3-P2-FC256-FC10	16 bit	155,951	233,516	282	1,794
(Zhang et al. 2021)	16C3-16C3S2-16C3S2-F10	8 bit	87,172	147,832	32	74
(Ye et al. 2023)	32C3-P2-32C3-P2-F256-F10	16 bit	80,172	138,658	245.5	/

* *xCy*(*Sn*) indicates that the layer is a convolutional layer containing *x* convolutional kernels of size *y*×*y* and the stride is *n* (if not marked, it is 1); *Py* indicates that the layer is a pooling layer with *y*×*y* size; *Fx* indicates that the layer is a fully connected layer or output layer containing *x* spiking neurons. The bold values in this table are only used to highlight the experimental data corresponding to the work of this paper, with no other special meanings.

### 5.2 Testing and validation for large residual networks

#### 5.2.1 Experimental setup

**Figure 14:**
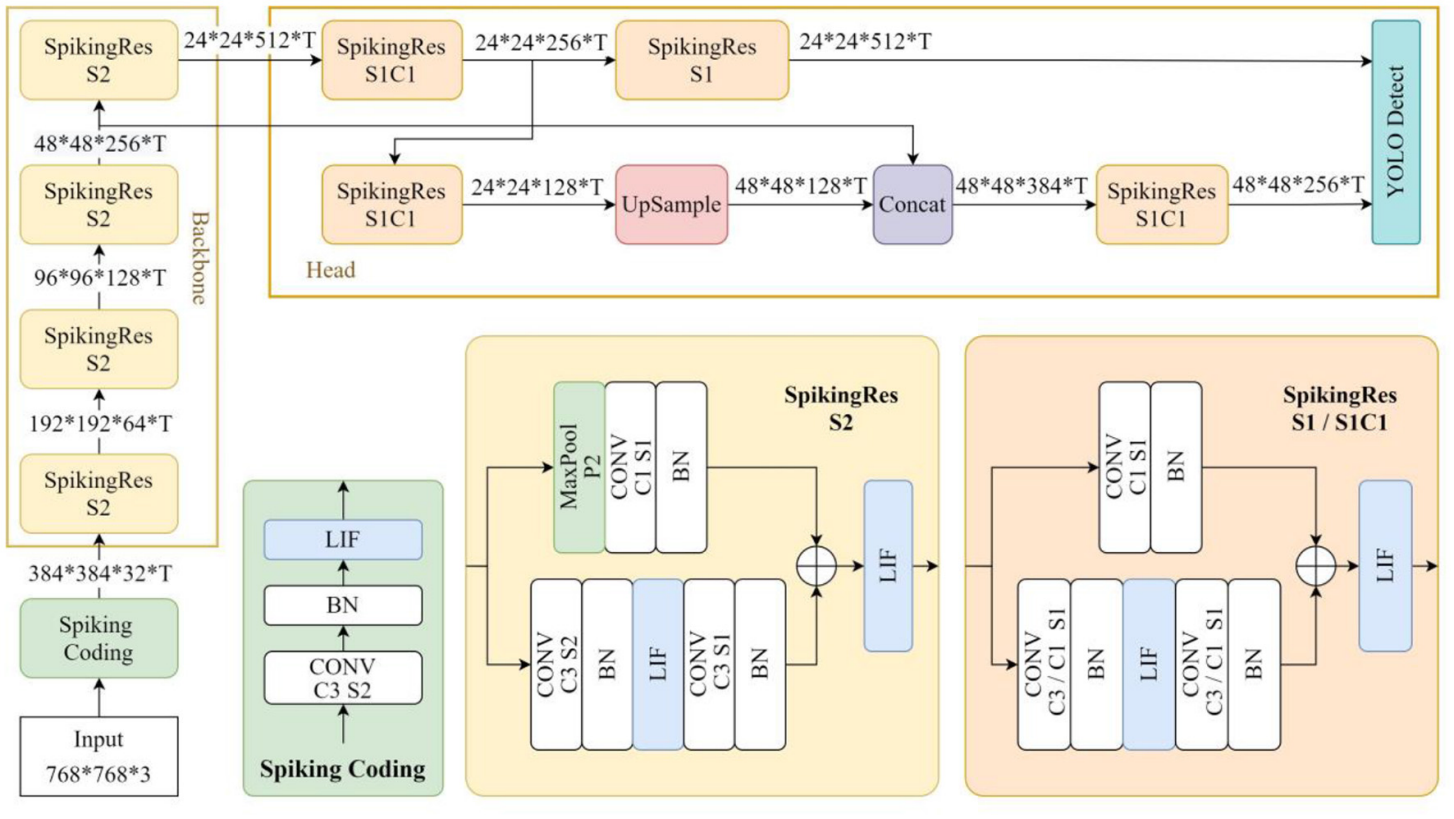
The network structure of SpikingRes-YOLO.

In this section of experiments, we designed a residual SNN called SpikingRes-YOLO for object detection tasks as the network model for this experiment. The structure of this model is shown in Figure 14. This model references the design ideas of the existing research called EMS-YOLO (Su et al., 2023), which successfully combines residual SNN with the YOLO framework to achieve energy-efficient object detection. We simplified the residual block of EMS-YOLO by combining the traditional residual structure, and then designed SpikingRes, a residual block in the form of full-spike input and output, to make it easier to realize in hardware. Based on this network, we verified the applicability of the proposed configuration and scheduling strategies to complex networks. The configuration strategies for different SpikingRes modules are shown in Table 5. The acceleration strategy for the entire network is based on the layer-by-layer reconfigurable computation for deep SCNNs to accelerate each SpikingRes module in the network one by one. When accelerating modules with larger feature map sizes, a line-by-line multi-timestep computation is used; for modules with wider networks, a grouped reuse computation is used; and for the remaining modules, a basic timestep pipeline computation method is applied.

**Table 5:** Configuration of each SpikingRes block in SpikingRes-YOLO.

**No**.	**Input feature map size**	**Output feature map size**	**Convolution layer**	**Weight matrix size**	**Computational strategy**
1	384*384*32 / 1,728 KB	192*192*64 / 864 KB	rconv0	32*32*3*3 / 9 KB	Line-by-line multi-timestep computation
			rconv1	32*64*3*3 / 18 KB
			sconv	32*64*1*1 / 2 KB
2	192*192*64 / 864 KB	96*96*128 / 432 KB	rconv0	64*64*3*3 / 36 KB	Line-by-line multi-timestep computation
			rconv1	64*128*3*3 / 72 KB
			sconv	64*128*1*1 / 8 KB
3	96*96*128 / 432 KB	48*48*256 / 216 KB	rconv0	128*128*3*3 / 144 KB	Grouped reuse computation
			rconv1	128*256*3*3 / 288 KB
			sconv	128*256*1*1 / 32 KB
4	48*48*256 / 216 KB	24*24*512 / 108 KB	rconv0	256*256*3*3 / 576 KB	Grouped reuse computation
			rconv1	256*512*3*3 / 1152 KB
			sconv	256*512*1*1 / 128 KB
5	24*24*512 / 108 KB	24*24*256 / 54 KB	rconv0	512*128*1*1 / 64 KB	Grouped reuse computation
			rconv1	128*256*3*3 / 288 KB
			sconv	512*256*1*1 / 128 KB
6	24*24*256 / 54 KB	24*24*512 / 108 KB	rconv0	256*256*3*3 / 576 KB	Grouped reuse computation
			rconv1	256*512*3*3 / 1152 KB
			sconv	256*512*1*1 / 128 KB
7	24*24*256 / 54 KB	24*24*128 / 27 KB	rconv0	256*64*1*1 / 16 KB	Timestep pipeline computation
			rconv1	64*128*1*1 / 8 KB
			sconv	256*128*1*1 / 32 KB
8	48*48*384 / 324 KB	48*48*256 / 216 KB	rconv0	384*128*1*1 / 48 KB	Grouped reuse computation
			rconv1	128*256*3*3 / 288 KB
			sconv	384*256*1*1 / 96 KB

* The feature map sizes in the table are computed based on the timestep *T* = 3, and the weight sizes are computed based on 8*bit* fixed-point numbers.

The network training was conducted on a workstation based on an Intel Core i9-14900K CPU and a NVIDIA RTX4090D GPU, using Pytorch and the SpikingJelly learning framework. For the LIF neuron model in the network, this section of the experiment uniformly sets the threshold voltage *V*_*th*_ to 1, the time constant τ to 2, and the parameter α to 1. Similarly, gradient descent based on the surrogate function was used for direct training.

**Figure 15:**
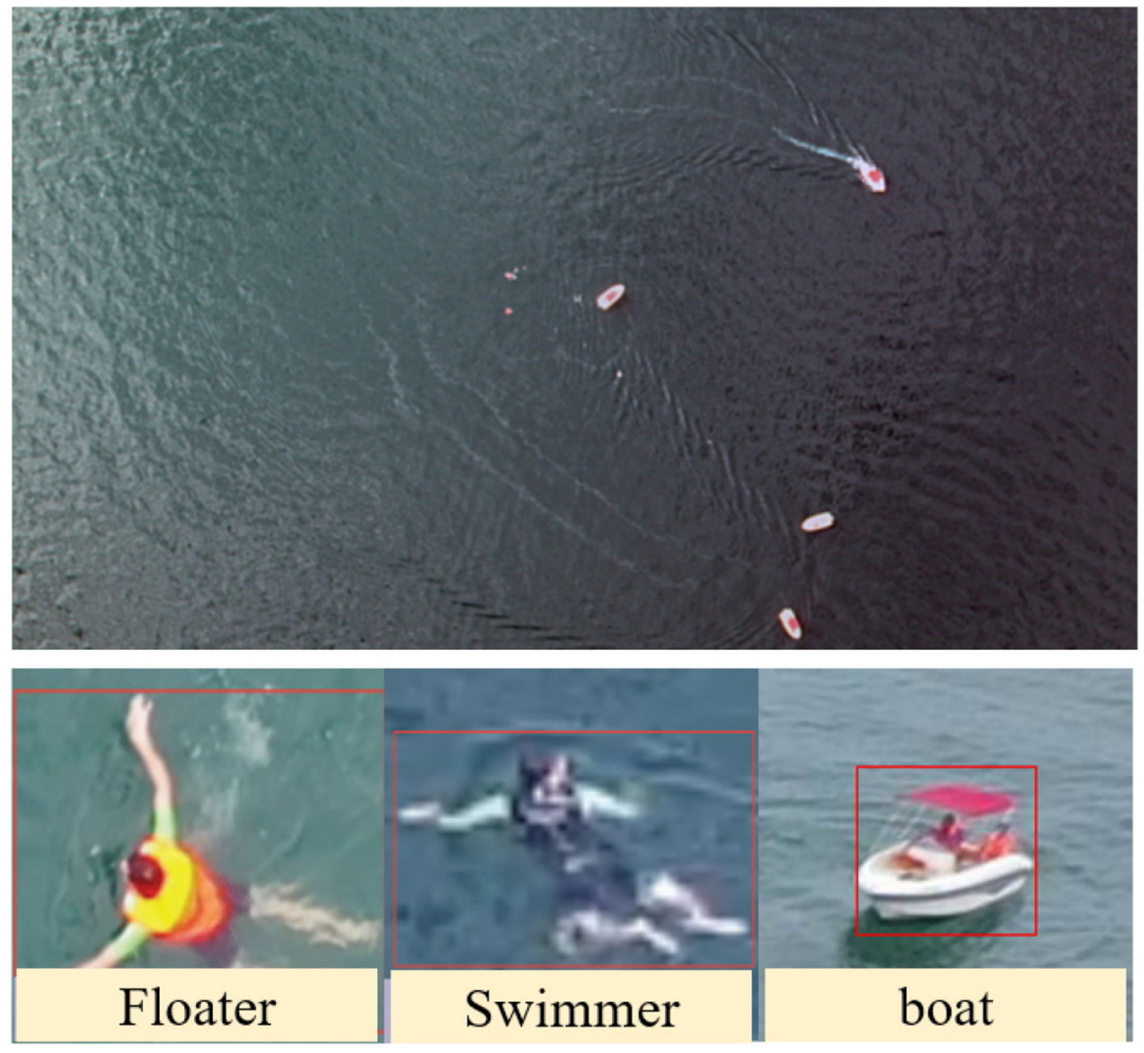
Examples of images and objects in the SeaDronesSee dataset.

The dataset used was the SeaDronesSee dataset (Varga et al., 2022), which aims to study the use of drones in maritime scenarios for search and rescue operations. The dataset consists of 5, 630 high-resolution aerial images captured by drones (some examples are shown in Figure 15), with a training set of 2, 975 images, a validation set of 859 images, and a test set of 1, 796 images, suitable for tasks such as object detection. This scenario has strict requirements for the power consumption and volume of hardware, and there is an urgent need for low-power object detection. It is an ideal application scenario for object detection algorithms based on SNNs. Considering the practical rescue needs in real-world scenarios, we mainly focused on the three categories of targets in the experiments: “boats", “floaters wearing life jackets in the sea", and “swimmers in the sea without life jackets" (as shown in Figure 15).

In this section of the experiment, the accelerator was configured with a ResNet structure. Its RTL codes were written in SystemVerilog and mapped onto an FPGA board, Xilinx VCU118, for synthesis and implementation. At the same time, the server based on CPU platform is also tested for processing the SpikingRes blocks as a benchmark. The server used had an Intel E5-2620 v4 CPU, with a clock speed of 2.1 GHz and a memory size of 141 GB. According to the specifications of each SpikingRes block, the number of parallel computing threads for each module was set to 128, and the timestep was set to 3. The trained weights were all stored in the DDR4 memory of the FPGA board, and the read and write operations of DDR were completed using the Xilinx Memory Interface Generator (MIG) IP. The quantization precision of the weights was set to 8-bit fixed-point numbers, including 1 bit for the sign, 3 bits for the integer part, and 4 bits for the fractional part. The clock frequency of the FPGA was set to 150 MHz. The weights of the batch normalization (BN) layer were fused with that of convolutional layer using the method in Zheng et al. (2021), so the accelerator did not need to process the BN layer during inference. Since the required timestep is very short and the neuron's membrane potential will be reset after exceeding the threshold voltage, only a 4-bit fixed-point number is used here to represent the intermediate state of the neuron (including 1 bit for the sign, 1 bit for the integer part, and 2 bits for the fractional part), to save space in the neuron state BRAM.

#### 5.2.2 Evaluation of experimental results

The effects of our accelerator on each SpikingRes block are shown in Table 6. It can be seen that for most of the SpikingRes blocks, the amount of operations is similar despite their different specifications. The running time of the accelerator for these different SpikingRes blocks almost corresponds to their operations amount. This indicates that the computational resources of the accelerator are fully utilized under these proposed computational strategies. The running time of each SpikingRes block is faster than that of the CPU, except for the first SpikingRes block, which takes longer than the other SpikingRes blocks due to the large image size and the small number of channels, which makes part of the computational resources unutilized.

**Table 6:** FPGA performance of accelerator on ResNet structure.

**SpikingRes No**.	**Computational strategy**	**Operations**	**Runtime of accelerator /ms**	**Runtime of CPU/ms**
1	Line-by-line multi-timestep computation	6.57 G	75.20	256.43
2	Line-by-line multi-timestep computation	6.57 G	38.08	143.73
3	Grouped reuse computation	6.57 G	37.58	94.38
4	Grouped reuse computation	6.57 G	38.32	65.79
5	Grouped reuse computation	1.70 G	20.08	29.07
6	Grouped reuse computation	6.57 G	38.31	62.78
7	Timestep pipeline computation	0.20 G	3.15	15.42
8	Grouped reuse computation	6.12 G	37.43	78.86
Average	/	/	36.02	93.31

And the overall comparison results of performance and power consumption between the accelerator and CPU platform are shown in Table 7. It can be seen that the average inference delay of the accelerator is 36.02 ms, i.e., the average framerate of each SpikingRes block is 27.76 FPS, which is 2.59 times that of the CPU platform. The measured power consumption of the CPU is 30 W; while the power consumption of the accelerator is 5.03 W, which is only 16.77% of that of the CPU. In terms of accuracy, the error of the hardware accelerator designed in this paper mainly comes from the fixed-point quantization of weights and neuron states. As can be seen from Table 7, under the condition of simulating by converting weights into 8-bit fixed-point numbers and neuron states into 4-bit fixed-point numbers, the network's accuracy loss is less than 0.01, which is still within an acceptable range.

**Table 7:** Performance and power consumption compared with CPU platform.

**Parameters**	**CPU (Baseline)**	**This work**
Platform	Intel E5-2620 v4	Xilinx VCU118
Clock Frequency	2.1 GHz	150 MHz
Bit width of weight	Float32	Fix8
Bit width of neuron states	Float32	Fix4
mAP@0.5	0.762	0.756
Power / W	30	5.03
Average runtime / ms	93.31	36.02
Average Framerate / FPS	10.71	27.76

The hardware resources consumed by the accelerator when mapped to the FPGA are shown in Table 8. The accelerator only uses 9.88% of the FPGA's hardware resources. The DSPs are only used in the calculation of BRAM addresses in the input buffer, and are not used in the process of spiking convolution, significantly reducing the demand for DSP resources.

**Table 8:** FPGA resource consumptions of accelerator on ResNet structure.

**Resource**	**Consumption quantity**	**Consumption rate**
LUT	71,443	6.04%
Register	53,777	2.27%
BRAM	669	30.97%
DSP	17	0.25%
Average	/	9.88%

Our accelerator is specifically tailored for edge scenarios, where deployment constraints (e.g., power budgets below 10W, compact form factors) make GPU-based solutions impractical. In contrast, edge devices (e.g., maritime drones in the SeaDronesSee task) often rely on CPUs as the baseline due to their low cost and compatibility with embedded systems—This is why we chose the CPU as the benchmark. Our accelerator achieves a usable frame rate of 27.76 FPS with a power consumption of 5.03W in such scenarios. The above experimental results demonstrate that the accelerator architecture and configuration scheduling strategy designed in this paper successfully achieve efficient hardware acceleration of a large residual topology SCNN model under limited hardware resources.

## 6 Conclusion

In this work, a multi-structure compatible and high-efficiency SCNN hardware accelerator architecture is proposed. This architecture completes SCNN operations by computing the convolution of input spikes at each timestep and updating neuron states. And it accelerates the computation by using an output channel parallelism and timestep pipeline architecture. The architecture supports both traditional convolutional topologies and residual convolutional topologies. Based on this accelerator architecture, we also propose configuration and scheduling methods such as grouped reuse computation and line-by-line multi-timestep computation, expanding the applicability of the accelerator to deep networks with large numbers of channels and large feature map size. The FPGA was used as the implementation platform for the accelerator and two scales of networks were configured to test and evaluate the accelerator, namely a small-scale LeNet and a deep residual SCNN for object detection. Experiments showed that the proposed architecture achieved a maximum MNIST recognition rate of 99.26% under the LeNet network, while achieving a speed of up to 1, 605 FPS and an energy consumption of only 0.65 mJ per image with reasonable resource usage; For residual modules in the deep residual SCNN, our architecture achieves a processing speed 2.59 times that of the CPU, while consuming only 16.77% of the CPU's power. Our work provides an efficient and flexible solution for the acceleration and deployment of SCNNs, offering broader prospects for the application and promotion of SCNNs in the future.

## Data Availability

The raw data supporting the conclusions of this article will be made available by the authors, without undue reservation.
